# Editorial: Women in pediatric cardiology 2021

**DOI:** 10.3389/fped.2023.1143383

**Published:** 2023-02-20

**Authors:** Laura Muiño-Mosquera, Sarah Nordmeyer

**Affiliations:** ^1^ Department of Pediatrics, Division of Pediatric Cardiology, Ghent University Hospital, Ghent, Belgium; ^2^ Department of Congenital Heart Disease - Pediatric Cardiology, Deutsches Herzzentrum der Charité - Medical Heart Center of Charité and German Heart Institute Berlin, Berlin, Germany

**Keywords:** myocarditis, exercise testing, fetal and maternal health, neurodevelopmental outcome, pediatric cardiology and surgery

Editorial on the Research Topic Women in pediatric cardiology 2021

Throughout the history of pediatric cardiology, multiple outstanding contributions have led to where this subspeciality stands today. In this editorial, we aim to review some of the extraordinary work of exceptional women in the field of congenital heart diseases and to collect current works led or performed by women in different fields of pediatric cardiology. In the early 30s, *Helen Taussig*- often referred as the mother of pediatric cardiology- established one of the first clinics for children with congenital heart defects and one of the first training programs for residents in pediatric cardiology in the United States (1). Her study on the “blue babies” and her idea on how to reconstruct a duct to relieve cyanosis in children with pulmonary valve atresia or critical stenosis led to the Blalock- Thomas-Taussig shunt, which, though modified, is still in use today. Two early fellows of Helen Taussig, *Mary Allen Engle* and *Ruth Whittemore*, also left an outstanding contribution by further establishing training programs in pediatric cardiology at Cornell and Yale Universities and being instrumental in the establishment of Pediatric Cardiology as a subspecialty at the America Academy of pediatrics (2). Dr. Whittemore was the recipient of the American Academy of Pediatrics Founders Award in 1995 for her study on the heritability in congenital heart diseases. In the early thirties, *Maude Abbott*, a Canadian pathologist, also published her “Atlas of Congenital Heart Disease” (3). Abbott's seminal work became crucial during the advent and progression of cardiac surgery.

With the development of new diagnostic techniques and new therapeutic options, survival of children with complex heart diseases increased tremendously; it became clear that congenital heart diseases required continuous follow-up into adulthood and that it was no longer a specialty dedicated to children. In 1975, *Jane Sommerville* established in London one of the first clinics for adults with congenital heart diseases worldwide. This initiative led to the birth of the Adult Congenital Heart Disease (ACHD) subspecialty (2), which is currently a recognized subgroup of the European Society of Cardiology (ESC). In addition to these great women, women like *Carolyn McCun*, *Jaqueline Noonan*, *Lynn Mahony*, or *Jane Newburger* have, in more recent years, made a great contribution to the field of pediatric cardiology. Writing about all of them would require a dedicated editorial.

With the increasing numbers of women in medical school and pediatric (cardiology) residency, the contribution to the field by women is rapidly increasing. Despite these high numbers of female students, the percentage tapers rapidly when looking at positions of higher responsibility. Women are still underrepresented in research and on editorial boards. As an example, a recent publication in JACC showed that only 19% of editorial board members among pediatric cardiology journals were women and all editors-in-chief were men (4). The causes of this discrepancy are multiple and fall beyond the scope of this editorial. This issue deserves a topic in itself.

In this special edition of Frontiers in Pediatric Cardiology, we collect current works led or performed by women in different fields of pediatric cardiology. It represents a glimpse of the latest not-yet-published work led by women in this field, although cannot reflect the vast amount of worldwide work performed by female researchers in pediatric cardiology.

## Heart failure and myocardial disease

*Al Wakeel-Marquard et al* show that an increased extracellular volume might be a non-invasive tissue marker of heart failure in patients with (congenitally corrected) transposition of the great arteries and systemic right ventricle. Although this study is limited by the amount of included patients (*N* = 13 and mild reduction of ventricular function- LVEF 51 ± 2%), it can trigger further study which can lead to the development of specific therapies to prevent heart failure in these patients.

*Seidel et al*. show in their manuscript that children with biopsy-proven myocarditis and lower levels of anti-β2-adrenergic receptor antibody, in comparison to the median, had a worse cardiovascular prognosis and less-event free survival. This finding requires further research but might help identify a group of children who might benefit from immunoglobulin therapy.

In their manuscript, *Thom et al.* identified that 72% of children with SARS-CoV2-associated multisystemic inflammatory syndrome present myocarditis with a good response to treatment with immunoglobulins and steroids.

*Brunet-García et al*, described for the first time that almost 90% of patients with congenital myotonic dystrophy type I present ECG anomalies, of which the most common are conduction disorders. Almost 2% of the patients in their cohort needed a pacemaker due to syncope in the context of progressive conduction disease and 7% of their patients died without a clear cause of death. This study highlights the importance of appropriate follow-up of arrhythmia and merits further study.

## Exercise physiology

Two of the studies included in this series focused on physical activity in Fontan patients and in patients affected by primary hypertension.

*Callegari et al.* show that only 18% of patients with Fontan circulation achieve the recommended 60 min/day moderate to vigorous physical activity. Physical activity, however, is positively correlated with physical well-being and mental health. Furthermore, it seemed to have a positive effect on sleep and behavior in this group of patients.

*Zhang et al.* describe impaired exercise capacity in children with primary arterial hypertension. Factors associated with decreased exercise capacity were female sex, younger age, greater BMI, and higher 24 h average diastolic blood pressure.

These studies suggest that appropriate training programs for adolescents with Fontan circulation or primary arterial hypertension might improve their physical performance and quality of life.

## Neurodevelopmental

The manuscripts of *Girch et al.* and Pfitzer et al*.* explore psychosocial and neurodevelopmental aspects of congenital heart diseases. Early hospitalization and interventions in children with congenital heart diseases are a source of stress which might influence their neurodevelopment. Family-centered care (FCC) has shown to reduce stress and improve healthy development in premature infants. The German EMPHATIC-30 questionnaire was previously used to evaluate FCC at neonatology and intensive care units. In Girch et al. the authors evaluate, for the first time, the psychometric properties of this questionnaire in children with congenital heart diseases.

*Pfitzer et al.* show that head circumference more than body weight and length might predict neurologic development and psychiatric problems in children with congenital heart diseases. Routine follow-up neurologic examination should be offered to all those children with congenital heart disease and especially to those at higher risk.

## Maternal and fetal outcome

In the manuscript of *Erba et al.* the authors describe maternal and fetal outcome in a large cohort (N = 307) of women with mechanical valves during pregnancy in Sudan. Due to the high cost of low Molecular weight heparin and the difficulty of regular measurement of anti-Xa levels, women with mechanical valves in Sudan are kept under an oral anticoagulant, warfarin, during pregnancy. Out of 307 pregnancies, only 47.6% had good maternal and neonatal outcomes. As a comparison, data from the European Society of Cardiology Registry of Pregnancy and Cardiac Disease (ROPAC) showed that 58% of the pregnancies included in the registry were uncomplicated (5).

*Seidl-Mlczoch et al.* focus on the outcome of fetuses with heterotaxy. Both left and right heterotaxia are associated with complex congenital heart defects and extracardiac problems. Termination of pregnancy rates are approximately 25% for both right and left isomerism. Postnatal survival is lower in those children presenting with right in comparison to left heterotaxy (46% vs. 67% at 5 years). This study confirms previous knowledge and helps prenatal counselling of fetuses with heterotaxy.

This special edition of Frontiers in pediatric cardiology collects a snapshot of the work led by female researchers in pediatric cardiology around the world. It shows the wide range of scientific fields, from myocardial and exercise physiology to neurodevelopmental and maternal and fetal outcome, which are covered by female scientists.

## Author contributions

LM-M has made a substantial contribution to the concept and design of the editorial and has approved the final version.

SN has made a substantial contribution to the concept and design of the editorial and has approved the final version.

## Conflict of interest

The authors declare that the research was conducted in the absence of any commercial or financial relationships that could be construed as a potential conflict of interest.
